# Gender-affirming robot-assisted sigmoid vaginoplasty: Outcomes and complications from a retrospective review of 12 cases

**DOI:** 10.1097/MD.0000000000047849

**Published:** 2026-02-28

**Authors:** Kyu Hyup Kim, Kyul Hee Kim, Na-Hyun Hwang, Min Jeong Kim, Hyun Cheol Jeong, Han Kyu Chae, Ji-Yeon Han, Jun Ho Park

**Affiliations:** a Department of Surgery, Kangdong Sacred Heart Hospital, Hallym University College of Medicine, Seoul, Republic of Korea; b Department of Plastic Surgery, Kangdong Sacred Heart Hospital, Hallym University College of Medicine, Seoul, Republic of Korea; c LGBTQ+ Center, Kangdong Sacred Heart Hospital, Hallym University College of Medicine, Seoul, Republic of Korea; d Department of Urology, Kangdong Sacred Heart Hospital, Hallym University College of Medicine, Seoul, Republic of Korea.

**Keywords:** gender-affirming surgery, robot-assisted surgery, sigmoid vaginoplasty, transgender women, postoperative complications

## Abstract

Gender-affirming surgery significantly improves the quality of life in transfeminine individuals. Among the various techniques available, sigmoid colon vaginoplasty offers both anatomical and functional advantages. However, conventional approaches are associated with high morbidity. Thus, we aimed to evaluate the outcomes of robot-assisted sigmoid vaginoplasty, emphasizing the complications and clinical results. Between July 2021 and March 2023, 12 patients underwent robot-assisted sigmoid vaginoplasty. Preoperative assessments included colonoscopy and abdominal computed tomography. Flap preparation and canal dissection were performed using a da Vinci XI platform. Postoperative management included vacuum-assisted closure dressing, bed rest, and vaginal dilator use. Clinical outcomes, complications, and sexual activity (SA) were retrospectively analyzed. The mean patient age was 27.9 years (95% confidence interval [CI]: 21.8–34.0). The mean total operative time was 544.8 minutes (95% CI: 494.4–595.1). Intraoperative complications were not observed. The mean vaginal length achieved was 14.6 cm (95% CI: 12.9–16.3), with no cases of vaginal stenosis. Positive SA was reported in 75% of the cases. The overall postoperative complication rate was 41.7% (5/12). Complications included ileus (2 cases, Grade II), vaginal prolapse (1 case, Grade IIIa), anastomotic leakage (1 case, Grade IIIb), and colon flap necrosis (1 case, Grade IIIb), all of which were successfully managed. Robot-assisted sigmoid vaginoplasty demonstrated favorable functional and aesthetic outcomes with manageable complications. However, anastomotic leakage and flap necrosis highlight the importance of careful consideration of vascular supply. Further comparative studies are required to evaluate its advantages over peritoneal vaginoplasty.

## 1. Introduction

Gender-affirming surgery (GAS) plays an essential role in the comprehensive care of transgender and gender-diverse individuals seeking to align their physical appearance with their gender identity. Among these procedures, vaginoplasty is particularly transformative for transfeminine individuals, alleviating gender dysphoria and significantly improving quality of life, self-esteem, and psychological well-being.^[1,2]^ The penile inversion technique remains the most commonly used method for vagina creation, but it is not suitable for every patient. Anatomical limitations, prior surgeries, or insufficient genital skin owing to prolonged hormonal therapy may necessitate alternative approaches.^[3–5]^

The introduction of the sigmoid colon for vaginoplasty in the 20th century marked a significant advancement, leading to refinements in surgical techniques over time. This approach has several advantages, including a natural mucosal lining that mimics vaginal secretions, a robust vascular supply, and sufficient length to create a functional and aesthetically pleasing vagina.^[6]^ However, traditional open and laparoscopic approaches to sigmoid colon vaginoplasty are associated with significant morbidity, including prolonged recovery times and complications such as anastomotic leaks, bowel obstruction, and vaginal stenosis.^[5,7,8]^

Recent advancements in robot-assisted surgery have provided new opportunities to enhance the precision, safety, and recovery of complex surgical procedures. These techniques offer improved visualization, greater dexterity, and the ability to perform minimally invasive procedures, leading to reduced postoperative pain and hospital stays (HSs) compared with open techniques.^[9]^ As a result, robot-assisted sigmoid colon vaginoplasty has become an increasingly appealing alternative for both surgeons and patients. However, the use of robotics in GAS remains relatively new, and limited data exist on long-term outcomes, patient satisfaction, and complications.^[10,11]^ In this study, we aimed to report our initial experience with robot-assisted sigmoid vaginoplasty, examine the associated complications, and retrospectively evaluate clinical outcomes.

## 2. Methods

### 2.1. Patients

**Figure 1.**
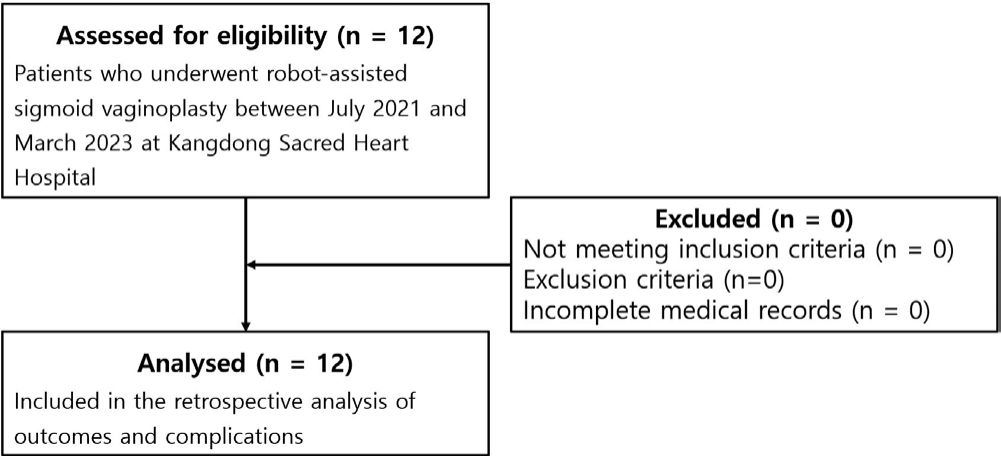
Patient flow diagram. A total of 12 patients were assessed for eligibility, and all were included in the final analysis without exclusion or loss to follow-up.

Robot-assisted sigmoid vaginoplasty was performed on 12 patients between July 2021 and March 2023. A flow diagram illustrating the patient selection process is presented in Figure 1. The inclusion criteria were transgender women aged 18 years or older diagnosed with gender dysphoria who desired vaginal reconstruction. Patients with a history of major abdominal surgery that prohibit the use of a sigmoid flap (SF), active inflammatory bowel disease, or incomplete medical records were excluded. Prior to surgery, all patients underwent comprehensive medical, psychological, and hormonal assessments, as well as colonoscopy and abdominal computed tomography (CT). This study was approved by the Institutional Review Board of Kangdong Sacred Heart Hospital on February 19, 2025 (IRB#2025-02-006). Sample size: This retrospective case series included all consecutive patients who underwent robot-assisted sigmoid vaginoplasty at our institution during the study period (July 2021 to March 2023). No patients were excluded from analysis, and no patients were lost to follow-up (FU). The sample size of 12 patients represents the complete institutional experience with this surgical technique during this timeframe. Written informed consent was obtained from the patient for the use of clinical photographs.

### 2.2. Preoperative preparation

We evaluated the abdominal and perineal CT scans and assessed the condition of the sigmoid colon using a colonoscope. All patients received electrolyte-based laxatives the day before surgery and underwent appropriate skin preparation.

### 2.3. Surgical procedure

**Figure 2.**
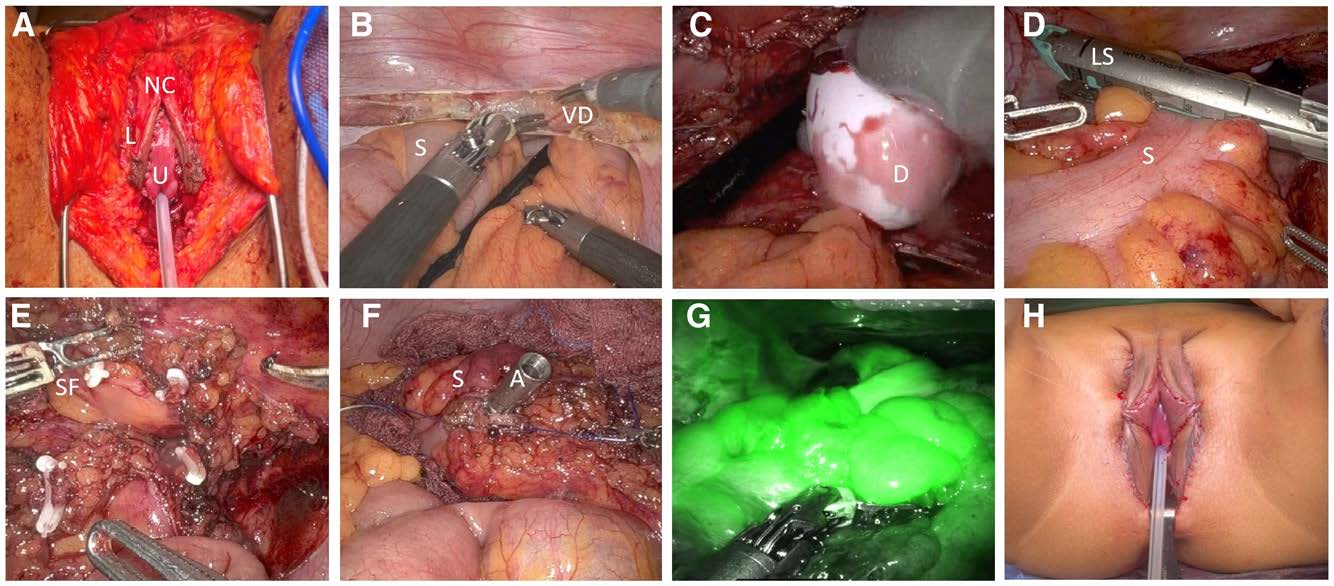
Procedures of robot-assisted sigmoid vaginoplasty. (A) Perineal view after orchiectomy, penile disassembly, and part of the vulvoplasty, including neoclitoris creation and labial reconstruction; (B) rectovesical dissection; (C) creation of vaginal space. A dilator inserted from the perineum is visible in the pelvic cavity; (D) sigmoid colon transection using a linear stapler; (E) division of inferior mesenteric and sigmoid arteries showing the sigmoid flap; (F) accommodation of the anvil within the sigmoid colon in preparation for anastomosis; (G) fluorescence imaging (Firefly™) of the colon flap; the green color indicates well-perfused tissue; (H) postoperative photograph of the genitalia. Images (B–G) are intra-abdominal views toward the pelvis. A = anvil, D = dilator, L = labium, LS = linear stapler, NC = neoclitoris, S = sigmoid colon, SF = sigmoid flap, U = urethra, VD = vas deferens.

Sigmoid vaginoplasty and external genital reconstruction teams performed all procedures. After the genital reconstructive surgery team prepared the perineal space, the robot-assisted surgery team proceeded to the colon flap preparation stage. Orchiectomy, penile disassembly, and part of the vulvoplasty, including neoclitoris creation and labial reconstruction, were performed. After the external genital structures were prepared (Fig. 2A), the transabdominal portion of the procedure was performed using the da Vinci XI platform (Intuitive Surgical, Sunnyvale). Intraperitoneal access was established using a standard approach, followed by robotic docking. Flap creation and canal dissection began with a horizontal incision in the peritoneum, covering the seminal vesicles within the rectovesical pouch (Fig. 2B). A dilator inserted from the perineum confirmed the position while a vaginal space was created between the rectum and prostate (Fig. 2C). The sigmoid colon was dissected from the lateral side, adhering to the principles of total mesorectal excision to achieve free mobilization. 15 cm segment was generally identified using a nylon tape, followed by blunt dissection while preserving the mesocolon. Proximal and distal transactions were performed using a robotic linear cutting stapler (Intuitive Surgical, Sunnyvale) to obtain a sigmoid colon flap (Fig. 2D). The inferior mesenteric artery (IMA) was cut off along with partial resection of the sigmoid arteries to enable mobilization of the flap to the perineum (Fig. 2E). An intracorporeal purse-string suture was created at the distal end of the sigmoid colon, and the anvil of the end-to-end anastomosis (EEA) stapler was accommodated (Fig. 2F). Intracorporeal end-to-end colorectal anastomosis was performed using an EEA stapler (Ethicon, Somerville) through the anus and the rectum. Real-time blood flow in the colon flap was assessed using near-infrared fluorescence imaging with the da Vinci Firefly™ Imaging System (Intuitive Surgical, Sunnyvale) (Fig. 2G). The pedicled recto SF was transposed to the dissected perineal region in an inverted orientation and anastomosed in a circular manner to the skin flaps derived from the perineum, groin, or scrotal areas (Fig. 2H).

### 2.4. Postoperative management

Vaginal packing was performed using vacuum-assisted closure dressing, which was removed in the operating room on postoperative day 5 to assess the condition of the flap. All patients remained nil per os until postoperative day 2 and continued bed rest until postoperative day 5. The use of a vaginal dilator was recommended to prevent stenosis at the colocutaneous junction and to aid in pelvic floor muscle rehabilitation. Resumption of sexual intercourse was permitted 6 to 8 weeks after surgery.

### 2.5. Postoperative follow-up and outcome assessment

Patients visited the outpatient clinic for FU assessments at 1, 3, 6, and 12 months postoperatively and annually thereafter. At each visit, postoperative outcomes were assessed via physical examination, including vaginal depth measurement using a graduated dilator, speculum examination to check mucosal status, and patient interviews regarding sexual function. Postoperative complications were classified and managed according to the Clavien–Dindo system. Specific definitions were established as follows: postoperative ileus was defined as a transient impairment of bowel motility accompanied by abdominal distension or delayed passage of flatus or stool, confirmed by abdominal radiography demonstrating dilated bowel loops or air–fluid levels. Vaginal prolapse was defined as symptomatic protrusion of the neovaginal sigmoid mucosa beyond the introitus. Sigmoid flap necrosis was defined as irreversible ischemia characterized by dusky or black discoloration of the mucosa with loss of capillary refill. Anastomotic leakage was defined as a defect at the colorectal anastomosis site confirmed via endoscopy or CT.

### 2.6. Statistical analysis

The primary endpoint of this study was the overall postoperative complication rate, categorized according to the Clavien–Dindo classification. The secondary endpoints included operative time, length of HS, vaginal depth at FU, and postoperative sexual activity (SA). Continuous variables were presented as means with 95% confidence intervals (CIs) or medians with interquartile ranges, while categorical variables were presented as frequencies and percentages. Time-to-event analysis for postoperative complications was performed using the Kaplan–Meier method to estimate complication-free survival. All statistical analyses were performed using Python programming language (version 3.11; Python Software Foundation, Wilmington) using the SciPy library (version 1.11.1; SciPy Developers, https://scipy.org). A two-tailed *P* value <.05 was considered statistically significant for all analyses.

## 3. Results

**Figure 3.**
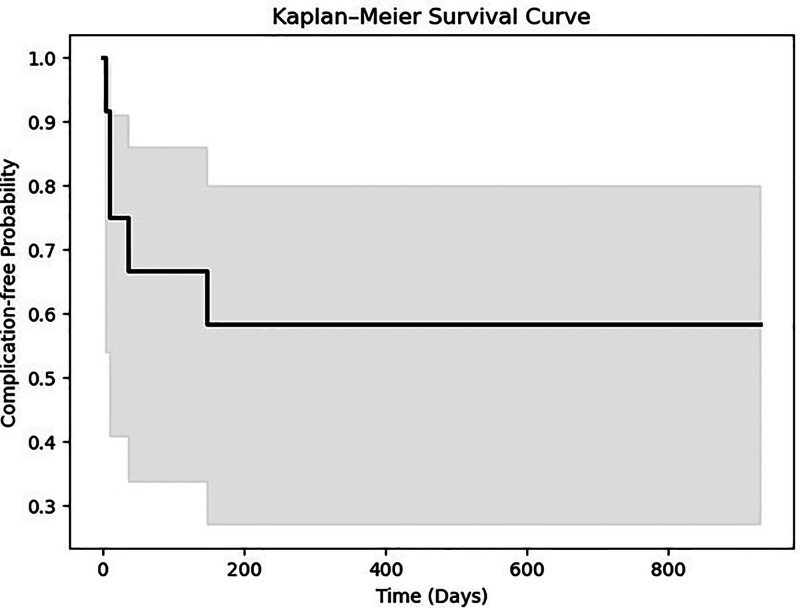
Kaplan–Meier estimate of complication-free survival. The curve illustrates the probability of remaining free from postoperative complications over time (d). The solid line represents the survival estimate, and the shaded area indicates the 95% confidence interval. The sharp drops represent the time points at which complications occurred.

Patient demographics, perioperative variables and surgical outcomes are presented in Table 1. The mean age of the patients at the time of surgery was 27.9 years (95% CI: 21.8–34.0), and the mean body mass index was 22.9 kg/m^2^ (95% CI: 21.2–24.7). The mean total surgery time was 544.8 minutes (95% CI: 494.4–595.1), with a mean docking time of 222.1 minutes (95% CI: 169.7–274.5). No intraoperative mortalities or complications were observed. The mean HS was 18.5 days (95% CI: 13.7–23.4), and the mean vaginal length was 14.6 cm (95% CI: 12.9–16.3), with no instances of vaginal stenosis observed. The median FU duration for the cohort was 14 months (interquartile range 12–25.5). Positive SA was reported in 75% (9/12) of the cases. Postoperative complications were observed in 5 cases. All complications were classified using the Clavien–Dindo system, and a summary of each event with corresponding management is provided in Table 2. Kaplan–Meier analysis (Fig. 3) demonstrated the timeline of these events, showing a cumulative complication-free survival rate of approximately 58% at the end of the FU period. Postoperative ileus developed in 2 instances and resolved after several days of fasting. Vaginal prolapse occurred in 1 case and was successfully managed through excision under local anesthesia. Additionally, anastomotic leakage was identified on postoperative day 10 in 1 case and treated with laparotomy, primary closure of the sigmoid colon leakage site, and creation of a loop ileostomy. Notably, leakage was not present with typical intra-abdominal peritonitis or pneumoperitoneum observed in standard anterior resections. Instead, it resulted in a fistula between the anastomotic site and perineal wound, which was not detected on abdominal CT but was confirmed endoscopically. Colon flap necrosis developed on postoperative day 6 in 1 case, necessitating debridement under general anesthesia on postoperative day 9. Daily aseptic dressing changes and additional debridement were performed, followed by application of skin and bilateral inguinal skin grafts on postoperative day 15. On postoperative day 29, the vaginal graft was intact, and the patient was discharged.

**Table 1:** Clinical characteristics and surgical outcomes of the 12 patients.

	Age	BMI (height/BW)	Medical history	Op-time (Console time), min	HS (d)	Complication	Vaginal depth (cm)	SA	FU (mo)
1	22	22.6 (165/62)	Arrhythmia	700 (420)	16		16.5	Yes	25
2	29	23.4 (172/69)		610 (312)	16		15	Yes	14
3	55	20.7 (166/57)		495 (185)	15	Ileus	16.5	Yes	12
4	26	25.0 (165/98)	Congenital cardiac abnormalities	560 (203)	15		14	No	26
5	18	25.4 (172/75)		460 (162)	13		15	Yes	10
6	28	19.7 (180/64)		520 (205)	17		15	Yes	12
7	25	23.8 (179/76)		525 (213)	15	Ileus	6.5	Yes	31
8	20	21.3 (162/56)		410 (135)	15		15	No	31
9	28	20.2 (167/56)		560 (175)	38	Anastomotic leakage	15	No	2
10	28	28.6 (162/75)		592 (195)	15	Vaginal prolapse	15	Yes	31
11	22	19.7 (171/57)		485 (155)	31	Sigmoid flap necrosis	16.5	Yes	14
12	34	25.2 (161/66)		620 (305)	17		15	Yes	16

BMI = body mass index, BW = body weight, FU = follow-up period (mo), HS = hospital stay, Op-time = operative time, SA = sexual activity.

**Table 2:** Summary of postoperative complications and management according to the Clavien–Dindo classification.

Case No.	Complication	Clavien–Dindo grade	Management
3	Ileus	Grade I	Conservative management with fasting and fluid therapy
7	Ileus	Grade I	Conservative management with fasting and fluid therapy
9	Anastomotic leakage	Grade IIIb	Laparotomy, primary repair of leak, creation of loop ileostomy
10	Vaginal prolapse	Grade IIIa	Excision of prolapsed mucosa under local anesthesia
11	Sigmoid flap necrosis	Grade IIIb	Debridement, bilateral inguinal skin grafts under general anesthesia, serial dressing

## 4. Discussion

**Figure 4.**
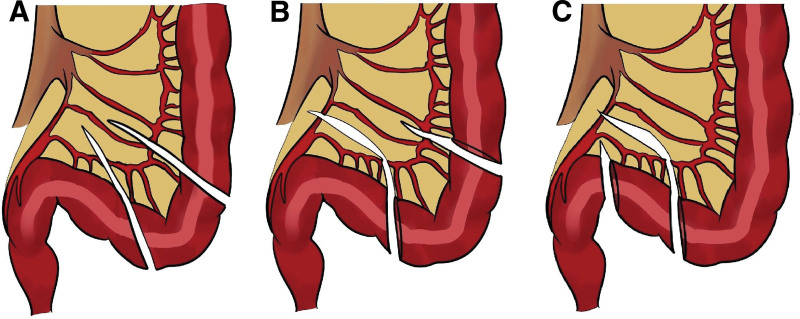
Vascular pedicle preservation design. (A) Preserving the blood flow the IMA to the SRA with partial resection of the sigmoid arteries; (B) superiorly based blood supply; (C) inferiorly based blood supply. IMA = inferior mesenteric artery, SRA = superior rectal artery.

GAS for gender dysphoria is becoming more prevalent as the recognition and acceptance of the condition increase.^[12]^ Vaginoplasty is a critical component of the male-to-female GAS. Various techniques have been explored, including penile inversion with autologous skin, sigmoid colon vaginoplasty, peritoneal vaginoplasty, and, more recently, methods employing autologous oral epithelial cells.^[13]^ Numerous studies have reported the diverse experiences and complications associated with this procedure (Table 3). A recent study by Sljivich et al in 36 patients demonstrated promising outcomes with introital stenosis occurring in 5.6% (2/36 cases requiring revision) compared to our 0% stenosis rate. However, our higher overall complication rate of 41.7% (5/12) compared to their reported series highlights the technical challenges and learning curve associated with this procedure. The differences may also reflect variations in complication definitions, FU duration, and reporting thresholds between studies.^[14]^ Despite these favorable findings, the clinically significant procedure-related risks remain. This report details our experience with gender-affirming robot-assisted sigmoid vaginoplasty, recording 5 postoperative complications in a series of 12 cases. Postoperative ileus developed in 2 cases and resolved after several days of fasting. Vaginal prolapse occurred in 1 case and was successfully treated with excision under local anesthesia. Previous studies have reported prolapse incidences of 11.1% and 17%.^[15,20]^ To prevent this complication, the perineal side of the rectosigmoid stump was secured, while the opposite side was laparoscopically pulled and sutured to the peritoneum under tension, gently drawing the vaginal skin into the sigmoid colon. Two cases of severe complications were noted. One case involved colonic flap necrosis that required reoperation under general anesthesia. The necrotic colon was debrided, and an inguinal skin graft was applied, leading to successful recovery. The literature on the blood supply to the colon flap describes 3 primary approaches^[15,16,18,19,21]^ (Fig. 4). One method preserves the blood flow from the IMA to the superior rectal artery with partial resection of the sigmoid arteries (Fig. 4A). Two techniques can be employed when resecting an IMA. One technique involves transecting the IMA distal to the sigmoid artery supplying the colonic flap, thereby preserving a superiorly based blood supply (Fig. 4B). The other technique involves transecting it proximal to the sigmoid artery, resulting in an inferiorly based blood supply (Fig. 4C). To achieve sufficient mobilization of the sigmoid colon flap for secure anastomosis with the perineal skin, an inferiorly based blood supply was used with the sigmoid colon flap inverted. In the present case of flap necrosis (Case #11), an inferiorly based blood supply with IMA division was utilized to achieve sufficient mobilization to the perineum, which may have contributed to suboptimal distal flap perfusion. Although no study has specifically investigated the relationship between necrosis and blood supply in sigmoid colon flaps, inferiorly based blood supply may provide less optimal flap perfusion than a superiorly based approach. In cases of congenital vaginal agenesis, particularly Mayer–Rokitansky–Küster–Hauser syndrome, the vaginal apex lies near the peritoneum, allowing adequate mobilization with only partial resection of the sigmoid arteries, without dividing the IMA. In contrast, GAS requires mobilization of the colon flap to the perineum which necessitate IMA division. Suboptimal perfusion of the colon flap may predispose patients to complications, such as stenosis or necrosis, highlighting the need for further investigation. Another severe complication, anastomotic leakage, required reoperation with ileostomy formation, followed by repair approximately 1 year later. The leakage resulted in a fistula between the colon and perineal operative site, confirmed by sigmoidoscopy in the absence of free intraperitoneal air or peritonitis. The incidence of colon anastomotic leakage ranges from 5 to 15% and can be life threatening in certain cases.^[22]^ Several studies on sigmoid vaginoplasty have reported leakage rates of 2 to 7%.^[15]^ Although limited by the small sample size, this study’s complication profile did not demonstrate any clear advantages over other laparoscopic approaches reported in the literature.^[7,15,16,18–21]^ However, a key advantage of the robotic approach is the ability to perform intracorporeal anvil placement for EEA, which eliminates the need for an additional 3 to 4 cm incision required in laparoscopic procedures. Postoperative complication rates reported in the literature range from 15 to 50% including ileus, wound infection, vaginal prolapse, urinary tract obstruction, voiding difficulty, introital stenosis, colovaginal fistula, SF necrosis, and anastomotic leakage.^[7,15–21]^ Although no direct comparative studies between robot-assisted sigmoid and peritoneal vaginoplasty are available, a study by Cao et al comparing laparoscopic sigmoid and peritoneal vaginoplasty in 40 cases revealed anastomotic leakage in the sigmoid group. By contrast, peritoneal vaginoplasty was associated with a less invasive approach, lower risk, superior satisfaction, and reduced morbidity.^[17]^ Yao et al found no significant differences in vaginal functional outcomes between laparoscopic sigmoid vaginoplasty and peritoneal vaginoplasty. However, the peritoneal approach was technically easier, did not result in bowel dysfunction, and carried a lower risk.^[8]^ Further studies on peritoneal vaginoplasty have demonstrated lower complication rates than those of sigmoid vaginoplasty. For example, Adam et al reported no complications in 40 cases of robot-assisted peritoneal vaginoplasty, while Dy et al observed a 13% reoperation rate in a study comparing da Vinci single-port versus Xi robot-assisted peritoneal vaginoplasty.^[23,24]^ Several sources of bias merit consideration. Selection bias is inherent to the retrospective, single-center design without a control group. Information bias was minimized through standardized physical examination protocols and structured patient interviews at predetermined FU intervals (1, 3, 6, and 12 months postoperatively, then annually). However, recall bias may affect self-reported outcomes such as SA. The absence of blinding in outcome assessment introduces potential detection bias, though this is difficult to avoid in surgical studies. Finally, the specialized nature of our LGBTQ+ center may introduce referral bias, as patients may differ systematically from those seeking care in general surgical settings. The findings of this study should be interpreted considering several limitations. The findings of this study should be interpreted considering several limitations. First, the retrospective and single-center design introduces potential selection bias that is inherent to such analyses. Second, the generalizability of these findings is limited by several factors. As a single-center experience from a specialized LGBTQ+ center in Seoul, South Korea, the results may not be representative of outcomes in other geographic regions, healthcare systems, or institutional settings with different volumes or surgical expertise. The small sample size (n = 12) and strict inclusion criteria further limit external validity. Additionally, the learning curve associated with this technically complex procedure may influence outcomes substantially in centers newly adopting this technique. These results should therefore be interpreted as preliminary institutional experience rather than definitive evidence of the technique’s general efficacy or safety. Third, the absence of a control group—such as patients undergoing conventional laparoscopic or peritoneal vaginoplasty – restricts the ability to determine the relative advantages among surgical techniques. Consequently, these results should be considered preliminary. Additionally, the heterogeneous FU duration (range 2–31 months, median 14 months) represents a limitation, particularly for detecting late complications such as stenosis or prolapse which may manifest months to years after surgery. Larger, multicenter studies with standardized protocols and long-term FU are warranted to validate these findings. In summary, although complications such as anastomotic leakage and SF necrosis underscore the risks associated with bowel-based vaginoplasty, robot-assisted sigmoid vaginoplasty remains an important option for patients with insufficient peritoneal tissue or specific anatomical constraints, particularly when meticulous vascular assessment and preservation are ensured.

**Table 3:** Comparative review of surgical complications in primary sigmoid vaginoplasty.

References	Cases	Surgery indication	Post-operative complications n (%)
Sljivich et al^[14]^	36	Gender dysphoria	Introital stenosis 2 (5.6)
Mukai et al^[15]^	18	Gender dysphoria	Anastomotic leakage 1 (5.6) Vaginal prolapse 2 (11.1)
Lenaghan et al^[16]^	60	Congenital vaginal agenesis	Anastomotic leakage 1 (1.6) Ileus 1 (1.6) Vagina rupture 2 (3.2) Pelvic peritonitis 2 (3.2) Introital stenosis 6 (9.6)
Cao et al^[17]^	14	Congenital vaginal agenesis	Anastomotic leakage 1 (7.1) Urinary retention 1 (7.1) Hydronephrosis 1 (7.1)
Bouman et al^[7]^	42	Gender dysphoria	Introital stenosis 6 (14.3) Postoperative bleeding 2 (4.7) Rectal perforation 1 (2.3) Mucosal prolapse 1 (2.3) Anastomotic leakage 1 (2.3)
Cai et al^[18]^	26	Congenital vaginal agenesis	Introital stenosis 2 (7.7) Ileus 1 (3.8) Wound infection 1 (3.8)
Salgado et al^[19]^	12	Gender dysphoria	Bowel obstruction 2 (16.7) Intraoperative bladder injury 1 (8.3) Deep vein thrombosis 1 (8.3) Pulmonary embolism 1 (8.3) Wound infection 1 (8.3)
Kim et al^[20]^	44 44 4	Gender dysphoria Congenital vaginal agenesis Cervical cancer	Defecation difficulty 7 (23) Mucus hypersecretion 5 (17) Introital stenosis 4 (13) Ileus 3 (10) Urination difficulty 4 (13) Vaginal prolapse 5 (17) Flap necrosis 2 (7)
Morrison et al^[21]^	83	Gender dysphoria	Protrusion 5 (6.1) Urinary obstruction 2 (2.5) Introital stenosis 16 (20) Rectovaginal fistula 2 (2.4) Urethral fistula 1 (1.2) Abdominal pain 2 (3.8) Ileus 3 (3.6) Other 8 (9.6)

## Acknowledgments

The authors thank Lina Park for helping with the illustrations.

## Author contributions

**Conceptualization:** Jun Ho Park, Kyul Hee Kim, Hyun Cheol Jeong, Han Kyu Chae.

**Data curation:** Kyu Hyup Kim, Jun Ho Park, Kyul Hee Kim, Min Jeong Kim, Han Kyu Chae, Ji-Yeon Han.

**Methodology:** Jun Ho Park.

**Supervision:** Na-Hyun Hwang, Hyun Cheol Jeong, Ji-Yeon Han.

**Writing – original draft:** Kyu Hyup Kim, Jun Ho Park, Na-Hyun Hwang.

**Writing – review & editing:** Hyun Cheol Jeong.
